# From tradition to translational medicine: advances and controversies in turmeric and curcumin research: Editorial on the Article Collection

**DOI:** 10.1080/13880209.2026.2743419

**Published:** 2026-10-11

**Authors:** Amy L. Roe, Igor Koturbash

**Affiliations:** a Procter & Gamble Healthcare, Cincinnati, OH, USA; b Department of Environmental Health Sciences, Fay W. Boozman College of Public Health, University of Arkansas for Medical Sciences, Little Rock, AR, USA; c Center for Dietary Supplements Research, Fay W. Boozman College of Public Health, University of Arkansas for Medical Sciences, Little Rock, AR, USA

Turmeric is obtained from the rhizome of *Curcuma longa* L. (Zingiberaceae) and has a long history of culinary, cultural, and medicinal use, particularly in South and Southeast Asia. Among its constituents, curcumin has received extensive scientific attention, while turmerones are major components of turmeric rhizome essential oil. During recent decades, turmeric and curcumin preparations have become widely available as dietary supplements and have been investigated for a broad range of biological and potential therapeutic effects. Interpretation of this literature is complicated by substantial differences in the botanical materials, extracts, isolated constituents, and formulations that have been studied. Product authenticity and composition, limited systemic exposure to native curcumin, and reports of rare liver injury introduce additional considerations when evaluating the efficacy and safety of these products. This Article Collection was launched to address these and related questions.

In the opening article, Upton provides an extensive ethnobotanical and pharmacognostic overview of turmeric, tracing its culinary, cultural, and medicinal uses and describing the botanical characteristics used to identify and authenticate *C. longa* rhizome (Upton 2026). The review surveys its historical use across Ayurvedic, Chinese, Persian, and Western herbal traditions, with particular emphasis on the extensive record from India, where digestive disorders and jaundice were among the recurrent traditional indications. The review also makes clear that turmeric and curcumin are not interchangeable terms: turmeric is a complex botanical material, whereas curcumin is one of its constituent curcuminoids and cannot be used interchangeably with turmeric when describing composition, exposure, or biological effects. Upton further summarizes macroscopic, microscopic, and chemosensory characteristics of the rhizome, together with post-harvest practices and common forms of substitution and adulteration that may affect the identity and quality of turmeric materials.

The extent to which marketed turmeric materials conform to their expected botanical and chemical composition was examined by Gafner and colleagues in a scoping review of 48 publications encompassing 2,235 commercial samples from six continents (Gafner et al. 2026). Across the included literature, 20.0% of samples were classified as adulterated, with similar proportions reported for spices and dietary or food supplements. The nature of adulteration differed between these categories. Spice products were reported to contain undeclared colorants, diluents, other plant materials, or other *Curcuma* species, whereas supplement adulteration more often involved synthetic curcumin or excessive dilution. The authors caution that these values provide only an estimate of adulteration because the available data are heterogeneous, sample numbers may not represent the entire market, and analytical methods were not always validated. When the analysis was restricted to reports using validated methods, the overall estimate decreased, while the proportion of samples classified as adulterated was higher among supplements than spices. The authors therefore emphasized that reliable authentication of commercial turmeric materials may require complementary analytical methods rather than reliance on a single assay.

Although much of the contemporary research on turmeric has focused on curcuminoids, the rhizome also contains a chemically distinct volatile fraction. Saavedra and colleagues characterize this fraction in authenticated and commercial turmeric oils and provide a detailed review of its safety and pharmacology (Saavedra et al. 2026). Ar-turmerone, α-turmerone, and β-turmerone were the dominant constituents, while differences in the broader chemical profiles reflected the source material and processing conditions. Comparison with commercial products also identified samples with compositions inconsistent with authentic turmeric rhizome oil, extending concerns about product identity and quality beyond curcuminoid preparations. The available toxicological studies were generally reassuring for the oils examined, whereas evidence for pharmacological activity remains predominantly preclinical. Human data are limited, and several clinical studies evaluated formulations containing both curcuminoids and turmeric oil, making it difficult to attribute observed effects specifically to the volatile fraction.

Gurley and colleagues examined how commercial formulation influences the release of curcuminoids and (S)-ar-turmerone under simulated gastrointestinal conditions (Gurley et al. 2026). Products representing different formulation strategies varied substantially in disintegration, dissolution, and bioaccessibility. Formulations marketed as providing enhanced bioavailability did not consistently demonstrate superior performance, while products with higher curcuminoid loads could generate greater absolute bioaccessible amounts despite relatively poor release efficiency. Thus, neither curcuminoid dose nor formulation strategy alone predicted the amount becoming bioaccessible under the conditions studied. The authors further emphasize that improved *in vitro* release is a prerequisite, but not a guarantee, for enhanced systemic exposure, which is additionally influenced by absorption, metabolism, and the chemical forms present in circulation.

Imtiaz and colleagues examined responses of lung and colorectal cancer cell lines to native curcumin at concentrations selected with reference to reported plasma data and compared them with higher concentrations commonly used in studies of curcumin cytotoxicity (Imtiaz et al. 2026). At the lowest concentration tested, curcumin did not significantly reduce cell viability and did not produce evidence of robust apoptosis. Nuclear Cyclin D1 levels increased in H460 and HT29 cells, decreased in A549 cells, and did not change significantly in Caco-2 cells. Marked loss of viability occurred only at the highest concentration. The authors concluded that responses depended on both concentration and cell line and that lower concentrations did not reproduce the cytotoxic effects observed at higher exposures. They also note that the lower concentration used in these experiments was selected with reference to upper reported total plasma exposure and should not be interpreted as an achievable concentration of free native curcumin. Following oral administration, circulating curcumin is predominantly present as glucuronide and sulfate conjugates, whereas unconjugated curcumin occurs at substantially lower concentrations. Cytotoxic effects observed at high *in vitro* concentrations therefore cannot be assumed to occur at the concentrations and chemical forms relevant to human systemic exposure.

The final contribution, by Akhtar and colleagues, addresses the safety of turmeric and curcuminoids, with particular attention to reports of hepatotoxicity associated with dietary supplements (Akhtar et al. 2026). The review was conducted by the United States Pharmacopeia Dietary Supplement Admission Evaluation and Labeling Expert Committee to determine whether the available evidence warranted cautionary language in the existing USP monographs. Animal toxicology studies generally indicated low acute toxicity and limited systemic toxicity for the preparations examined, although hepatic injury was reported in several studies at exposures substantially exceeding estimated dietary intake. Clinical studies were also largely reassuring: the reviews and trials evaluated by the committee reported no organ toxicity or serious adverse events, and the adverse effects observed were predominantly mild and transient. The authors nevertheless identified substantial limitations in this evidence, including heterogeneity in formulations, doses, and treatment duration, variable methodological quality, inconsistent biochemical monitoring, incomplete assessment or reporting of adverse events, and limited follow-up. As the authors note, clinical trials are well suited to identifying common adverse effects occurring during the period of observation but are less capable of detecting uncommon events or reactions with a longer latency.

The committee also considered spontaneous adverse event reports and published cases of clinically apparent liver injury. Spontaneous reporting systems contained reports of hepatobiliary events, which the authors treated as signals rather than estimates of incidence or evidence of causality, given that individual reports may be incomplete or duplicated and often provide limited information on exposure and other contributing factors. Published case reports and case series provided more detailed clinical information. The reported injury was predominantly hepatocellular, usually developed after approximately one to four months of use, and generally improved after discontinuation, although rare cases progressed to acute liver failure. Positive rechallenge strengthened causal attribution in several reports and reported RUCAM assessments ranged from possible to highly probable. Interpretation nevertheless remained limited by incomplete characterization of the implicated products, concomitant medications or supplements, alcohol use, and incomplete clinical information. The case literature therefore supports a rare hepatic safety signal associated with turmeric or curcuminoid supplements, while causal attribution in individual cases and identification of the responsible constituent or product characteristic often remain uncertain.

Causal interpretation is further complicated by incomplete understanding of the mechanism of injury and, in many cases, limited information on the composition of the implicated products. Akhtar and colleagues describe the reported liver injury as apparently idiosyncratic, with evidence suggesting a possible role for immune mechanisms (Akhtar et al. 2026). The HLA-B*35:01 allele has been identified in several cases, while increased curcuminoid exposure, mitochondrial dysfunction, and oxidative stress have also been proposed as possible contributors; none of these observations establishes a single mechanism applicable to all reported cases. Product quality represents a separate source of uncertainty. Adulteration, contamination, undeclared ingredients, and other constituents may contribute to some events, but most implicated products were not analyzed sufficiently to determine their composition independently. The possibility of adulteration therefore warrants investigation but cannot be assumed to explain reported liver injury in the absence of product analysis. Several regulatory and scientific bodies have concluded that the available evidence warrants liver injury precautions for turmeric or curcuminoid supplements. Mechanistic and predictive evidence can strengthen causal interpretation, while clinical recognition of a rare idiosyncratic reaction may precede identification of a fully defined mechanism.

Following its evaluation, the USP DSAEL EC recommended adding a cautionary statement concerning liver injury to the USP monographs for turmeric and curcuminoids. The cautionary statement notes that ‘Liver problems have been reported very rarely in people taking supplements containing turmeric and/or curcuminoids’ and advises individuals with a history of liver problems to consult a health care practitioner before use. It also recommends discontinuing the product and seeking medical advice if abdominal pain, dark urine, or jaundice develops (Akhtar et al. 2026. The recommendation for individuals with previous liver problems is precautionary. Likewise, the effect of such labeling on prevention of liver injury has not been determined. The cautionary statement therefore communicates the reported hepatic safety concern and provides guidance for recognition and response while retaining the uncertainty surrounding incidence and causal attribution in individual cases.

Interpretation of turmeric research requires precise definition of the botanical material and its chemical composition before biological or clinical findings can be attributed to turmeric, curcumin, or other constituents of *C. longa* (Upton 2026; Gafner et al. 2026). For formulated products, the administered or labeled amount cannot be considered independently of disintegration, dissolution, absorption, metabolism, and the chemical forms present in circulation (Gurley et al. 2026; Imtiaz et al. 2026). Experimental findings likewise require consideration of whether the concentrations and chemical forms tested can reasonably be related to human exposure. Controlled trials, spontaneous reports, clinical case reports, mechanistic studies, and product analyses contribute different information to safety assessment and have different limitations. Retention and chemical characterization of products implicated in adverse events would improve causal assessment, particularly when adulteration, contamination, or undeclared constituents remain possible (Akhtar et al. 2026; Gafner et al. 2026). The USP cautionary wording reflects this evidentiary balance by acknowledging that liver problems have been reported very rarely without stating that turmeric or curcuminoids generally cause liver injury (Akhtar et al. 2026). Future studies will be strengthened by authentication and chemical characterization of the materials examined and direct assessment of formulation and exposure.

## Data Availability

There is no data associated with this research.
